# A Systematic Review of Telemedicine for Older Adults With Dementia During COVID-19: An Alternative to In-person Health Services?

**DOI:** 10.3389/fneur.2021.761965

**Published:** 2021-12-14

**Authors:** Sasha Elbaz, Karin Cinalioglu, Kerman Sekhon, Johanna Gruber, Christina Rigas, Katie Bodenstein, Kamran Naghi, Paola Lavin, Kyle T. Greenway, Ipsit Vahia, Soham Rej, Harmehr Sekhon

**Affiliations:** ^1^McGill Meditation and Mind-Body Medicine Research Clinic and Geri-PARTy Research Group, Lady Davis Research Institute and Jewish General Hospital, McGill University, Montreal, QC, Canada; ^2^Temerty Faculty of Medicine, Toronto, ON, Canada; ^3^Douglas Mental Health University Institute, Montreal, QC, Canada; ^4^Department of Psychiatry, McGill University, Montreal, QC, Canada; ^5^Technology and Aging Laboratory, McLean Hospital, Boston, MA, United States; ^6^Harvard Medical School, Boston, MA, United States

**Keywords:** telemedicine, older adults, dementia, mental health, COVID-19

## Abstract

**Introduction:** Older adults with dementia have been significantly at more risk for not receiving the care needed and for developing further mental health problems during COVID-19. Although the rise in telemedicine adoption in the healthcare system has made it possible for patients to connect with their healthcare providers virtually, little is known about its use and effects among older adults with dementia and their mental health.

**Objective:** This systematic review aimed to explore the use, accessibility, and feasibility of telemedicine in older adults with dementia, as well as examine the potential mental health impacts of these technologies, through reviewing evidence from studies conducted during COVID-19.

**Methods:** PubMed, Scopus, and Web of Science databases were searched with the following keywords: (COVID^*^ OR SARS-CoV-2 OR Coronavirus) AND (“mental health” OR Depression OR Stress) AND (Dementia OR Multi-Infarct Dementia OR Vascular Dementia OR Frontotemporal Dementia) AND (elder OR Aging OR Aging OR Aged) AND (Telemedicine OR “Remote Consultation” OR telehealth OR technology).

**Results:** A total of 7 articles from Asia, Europe, and the United States were included in this review. Throughout the studies cognitive and mental health assessments (e.g., MoCA, FAST, etc.) were performed. Despite the barriers, telemedicine was noted as a feasible approach to assist individuals with dementia in connecting with their service providers and family while reducing complications related to travel (e.g., difficulty moving, traffic, distance).

**Conclusions:** Due to the COVID-19 pandemic, finding alternative ways to provide services to older adults with dementia through technology may continue to become more necessary as time goes on.

## Introduction

The COVID-19 pandemic rendered older adults more vulnerable to not receiving the healthcare needed and placed those living with dementia at an even increased risk for developing other mental health symptoms due to social isolation and loneliness (1). Telemedicine, an approach that incorporates information and communication technologies in the delivery of health care services for the diagnosis, treatment, prevention, and research and evaluation in order to advance patients' health outcomes, became more widely used following the dramatic rise in and the necessity for internet-based services during the COVID-19 pandemic (2). Telemedicine has proved a viable alternative in providing individuals with appropriate services and care along with mitigating against the effects of social isolation, especially in older patients with dementia [e.g., (3)].

Telemedicine is considered an effective option while reducing cost and increasing access to care in psychiatry treatment (4). According to a national poll released by the Canadian Medical Association (5), Canadians who connected with their doctor virtually during COVID-19 reported a high level of satisfaction (91%). Moreover, 46% of survey respondents who used virtual care would prefer a virtual method as a first point of contact with their doctor moving forward (5).

Although telemedicine is being more widely used as an effective and low-cost option, little is known about the impact of different telemedicine approaches on older adults with dementia and their mental health. Furthermore, the accessibility of telemedicine needs to be investigated as the use of beneficial technological alternatives to in-person health services may become more common post- COVID-19. This systematic review aims to explore the use, feasibility, and acceptability of telemedicine applications for older adults with dementia during the COVID-19 pandemic to address these gaps, as well as examine the potential mental health impacts of these technologies.

## Methods

### Search Strategy and Keywords

This systematic review only included research articles from 2020 to October 2021. Relevant keywords and *Medical Subject Headings* (MeSH) were identified and searched through 3 databases: PubMed, Scopus, and Web of Science Core Collection. Quantitative, qualitative, and mixed-method articles were included. We applied the following filter within all databases: “English”. In PubMed, we applied our search string within the query box marked with “All Fields”. In Scopus, we applied our search string in the query box with “Article title, Abstract, Keywords”. In Web of Science, we entered our search string in the query box with “All Fields” and we applied the following filter: “Open Access”.

### Search Criteria

PubMed, Scopus, and Web of Science databases were searched with the following keywords: (COVID^*^ OR SARS-CoV-2 OR Coronavirus) AND (“mental health” OR Depression OR Stress) AND (Dementia OR Multi-Infarct Dementia OR Vascular Dementia OR Frontotemporal Dementia) AND (elder_ OR Aging OR Aging OR Aged) AND (Telemedicine OR “Remote Consultation” OR telehealth OR technology). The total number of number of articles across all 3 databases was 83 (PubMed: 28, Scopus: 26, and Web of Science Core Collection: 29). We also examined the references of articles to ensure we did not exclude any relevant articles (i.e., snowballing).

### Selection of the Studies

Two independent reviewers (S.E.; K.C.) examined the articles and consulted with the senior researcher in the case of uncertainty (H.S.). From the combined total of the 3 databases, 83 articles were identified from the databases, of which 24 duplicates were removed and 1 article was identified via snowballing. Next, 39 articles were removed based on the titles not relating to the topic. We then assessed the abstracts of 21 articles, in which we applied the following exclusion criteria: (1) did not include older adults (60+) [3 article removed], (2) mental health outcomes were not clearly described [0 article removed], (3) the study was not conducted with individuals with dementia [2 articles removed], (4) no original data [9 articles removed], (5) telemedicine was not the primary focus of the study [0 article removed], (6) article was not based in the COVID-era [0 articles removed] (total n removed = 14). The resulting are the 7 published works included in this systematic review.

## Results

### Overview of Studies Included

**Figure 1:**
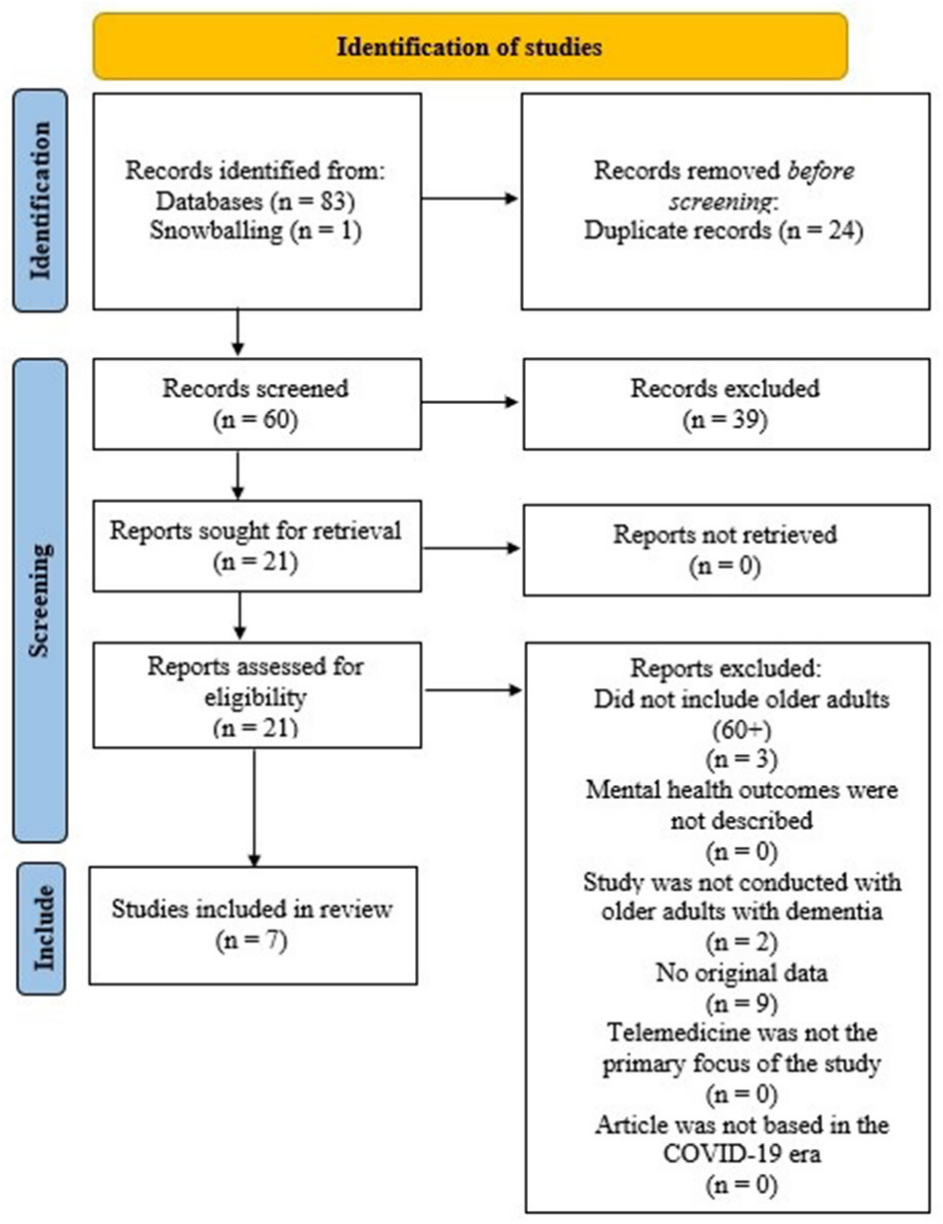
PRISMA flow diagram illustrating the selection of the studies.

The Preferred Reporting Items for Systematic Reviews and Meta-Analyses (PRISMA) flow diagram illustrating the selection process of the studies is presented in Figure 1 (6). An overview of each of the seven articles included is provided in Table 1, including details on authorship, study design, the sample population, demographics, use of a control group, the aim of each study, assessments used, and important findings. Overall, the geographic locations of the studies included four studies conducted in Europe (7, 9, 10, 12), two in the United States of America (8, 11), and one in Asia (3). Most of the research articles focused on recruiting community-dwelling participants (3, 9–11), whereas the others recruited participants through convenience samples in clinics, hospitals, care homes, and day centers (7, 8, 12). All studies were conducted within the COVID-19 context.

**Table 1:** Summary of the studies (*n* = 7).

	**References**	**Study design**	**Sample**	**Demographics**	**Population**	**Control**	**Aim/objectives**	**Other**	**Assessments**	**Conclusions**
1)	Arighi et al. (7)	Cross sectional/ Longitudinal	108 (>70 years)	51.4% with successful televisit 41.2% male with failed televisit	Patients from the Alzheimer's Center of the Fondazione IRCCS (Italy)	No control	To examine the issues with access to/use of digital technology (i.e. digital divide) in older adults with dementia contacted through videoconferencing		Remote neurologist consults/ interviews, MMSE	68.5% (74 patients) successfully connected via televisit 31.5% (34 patients) failed to respond to the televisit Failure to respond to televisiting due to connection difficulties do not access to devices/Internet Presence of young caregiver significantly influences televisit success (*p* <0.001, OR 5.14).
2)	Iyer et al. (8)	Longitudinal	43 (*M*_age_ = 85.5 years)	72.1% had degree of cognitive impairment	Older adults that receive services from an academic outpatient geriatrics clinic (USA)	No control	To examine the feasibility and acceptability of telemedicine visits in clinic serving older adults with a high proportion of cognitive impairment		Face-to-face or phone calls interviews 7-question optional experience survey for patients or caregivers 4-question survey for clinical providers	Patients and clinicians responses did not differ in *similarity of in-person visit* (*p* =0.999). Patients indicated greater comfort with using video or telephone visits in the future Telemedicine services are appreciated for frail, older adults
3)	Lai et al. (3)	Longitudinal	60 [30 control and 30 intervention] *M*_age_ patients with NCD = 72.73 ± 0.84 years) (*M*_age_ Caregivers = 71.83 ± 0.80 years	21 patients in the control group received between 4- 8 hours of support by family, 9 received > 8 hours of support 15 patients in the intervention group received between 4- 8 hours of support by family, 15 received > 8 hours of support	Convenience sample of community-dwelling people with cognitive impairment and spousal caregivers through an activity day center for older adults (China)	Control	To evaluate the extent to which both telehealth videoconferencing and regular telephone calls would provide benefits to older adults with NCD and their caregivers during COVID-19	Older adults with NCD presenting with major physical disabilities, such as strokes were excluded	Weekly telephone calls/ Weekly health services via Zoom, WhatsApp, or Facetime. Validated Chinese versions of MoCA, RMBPC, QoL-AD SF-36v2; ZBI scale, RCSES	Additional telemedicine had a significant impact halting the reduction of MoCA scores that was shown in the telephone-only group (η2 = 0.50). Improvements in physical and mental health of caregivers in the video-conferencing group but not telephone-only (η^2^ = 0.23–0.51).
4)	Goodman- Casanova et al. (9)	Cross-sectional Survey Part of a larger RCT	93 (*M*_age_ = 73.34 years)	65% of the sample were women 74% were living with other people	Community dwelling older adults with mild cognitive impairment/mild dementia recruited through convenience sample by the Biomedical Research institute of Malaga (Spain)	Control	To explore the impact of confinement on the health and well-being of community-dwelling older adults with mild cognitive impairment or mild dementia To provide television-based and telephone-based health and social support To evaluate a television-based technology for older adults with various forms of cognitive decline	Older adults with a score of > 11 on the GDS, terminal illness, and Individuals with cognitive, visual, motor conditions that could affect the system were excluded.	GDS MMSE Telephone based survey with open ended (qualitative) and numerically based (quantitative) questions administered by health professionals	No significant differences between intervention and control groups across all study variables (*p* > 0.05) Participants with TV-AssistDem did perform more memory exercises than the control group (*p* <0.001).
5)	Zeghari et al. (10)	Observational cross-over	8 (*M*_age_ = 76.7 years)	4 men; 4 women	Community dwelling participants that are socially isolated (France)	No control	To evaluate the feasibility and reliability of mobile unit settings for remote cognitive testing	Individuals with significant vision and auditory problems which would impact ability to perceive and understand the clinician were excluded	Short clinical interview, cognitive screening tests, Acceptability scale, Two versions of MMSE, FAB, 5 words: 5 mots de Dubois; SVF; PVF; DS	No significant differences between in-person testing vs mobile testing (*p*s *=* 0.115–1) Acceptability scores revealed that all participants found the MU easy to access and as comfortable ass being face-to-face
6)	Gately et al. (11)	Cross-Sectional	24 (range_age_ = 45 - ≥ 75)	Veterans with dementia (100% Male) All participants were white. One caregiver had prior experience with teleconferencing services for dementia management, all caregivers had experience with video conferencing	Community-dwelling caregivers of Veterans with Dementia (USA)	No control	To evaluate the role of in-home video telehealth technologies to meet the needs of caregivers and persons with dementia To identify strategies to adapt in-home video telehealth services		Semi-structured qualitative interviews (approx. 20 minutes long)	Caregivers describe that telehealth services can be beneficial as a follow-up service Caregivers propose that one barrier technological implementation for older adults with dementia is that they may have limited ability to engage/ manage the devices without help
7)	Zamir et al. (12)	Collaborative action research (CAR)	22 older adult residents (≥ 65 years) 8 facilitators (22–50 years)	7 residents with dementia or signs of cognitive decline 12 residents with hearing impairment 9 with visual impairment 3 that are non-verbal 6 that are frail	Convenience sample of older adults in care homes (UK)	No control	To explore the feasibility and accessibility of whether video-calls between care homes could reduce loneliness and social isolation in older adults.		Ethnographic approach consisting of observations, informal unstructured feedback, memo writing and semi-structured interviews	Five dominant themes were revealed Some residents living in the care home seemed to have regained their energy and self-purpose because of the video calls. Therefore, the increase of the residents' social networks by connecting them to other care home residents may have helped decrease their loneliness

Overall, only two of the seven articles included any form of control group or condition, which included participants receiving either a phone call or reduced telemedicine service (control) as compared to receiving the intervention in its full form [i.e., (3, 9)]. One of the studies, which used a control, utilized a cross-sectional survey design in which both the intervention and control groups had to respond to a telephone-based survey (9), whereas the other study had participants divided between a weekly phone call group and a weekly phone call plus video calling group (3).

Among the seven articles, five did not use control groups. Instead, the researchers performed longitudinal, cross-over, cross-sectional, or collaborative action research designs. Articles 2 (8), 5 (10), and 7 (12), from Table 1, investigated a number of medical clinics that were in the process of examining the feasibility and implementation of technological interventions for telemedicine services. Articles 1 (7) and 6 (11), as identified in Table 1, evaluated the roles of technology in older adults with memory decline and dementia and explored the potential barriers to technology use that individuals with cognitive decline or impairment may experience. Finally, the researchers attempted to test the benefits of emerging technologies in the third (3) and fourth (9) articles. Specifically, article 3 evaluated the beneficial impacts of phone calls or phone calls plus supplementary video calling (3) and article 4 evaluated a television-based technology (9).

Several different tests and assessments were used in the studies, which included measures of mental state or cognitive ability, experience, accessibility, etc. All studies included participants aged 65+. The majority of these studies, excluding Gately et al. (11), had both male and female participants. With regards to age, all studies, apart from Zamir et al. (12), conducted their work with adults aged at least 70 years and older, whereas the work by Zamir et al. (12) included participants aged 65 and over.

Various studies screened participants prior to the consultations. Several factors were screened for prior to participant enrolment in the respective studies, such as physical disabilities like strokes, terminal illnesses, visual impairment, motor impairment, auditory impairment, and negative affect (using the Geriatric Depression Scale [GDS]). Lai et al. (3) screened their participants to exclude individuals with a history of strokes. Goodman-Casanova et al. (9) conducted pre-screening for participants with motor, cognitive, and visual conditions that could affect the participants' use of the television-based technology. Zeghari et al. (10) also evaluated the feasibility and reliability of a mobile unit for cognitive testing and pre-screened individuals to ensure they were not presenting with significant visual or auditory limitations that could impact their participation.

### Cognitive and Mental Health Outcomes

The studies used various cognitive tests, including the Mini-Mental State Examination (MMSE), Montreal Cognitive Assessment (MoCA), the Quality of Life in Alzheimer's Disease (QoL-AD) scale, Geriatric Depression Scale (GDS), etc., to assess the effectiveness of telemedicine. One study found significant differences in the MoCA scores of individuals with cognitive impairment receiving both telephone and video calls compared to the telephone service-only group (12). Comparatively, two other studies found no significant differences between their 2 groups, albeit with some discrepancies (9, 10).

Lai et al. (3) found that individuals receiving additional telemedicine video calls had significantly higher MoCA and quality of life (demonstrated through the QoL-AD) scores [MoCA: *F*_(1, 58)_ = 17.97, *p* < 0.001, η^2^ = 0.24; QoL-AD: *F*_(1, 58)_ = 5.54, *p* < 0.05, η^2^ = 0.09]. It was also remarked that within the 4-week period within which the study had begun, the control group's (receiving telephone calls only) MoCA scores fell by 1.83 points (3). Goodman-Casanova et al. (9), however, found no significant differences between their intervention group and control groups across all study variables, which included the GDS, MMSE, and an in-house developed survey. Comparatively, Arighi et al. (7) found no significant differences across MMSE scores from previous in-person visits to online teleconsultations. Moreover, Zeghari et al. (10) found no statistically significant differences between face-to-face and mobile testing, using two different versions of the MMSE, FAB, SVF, etc. This finding by Zeghari et al. (10) is noted as an important indicator for future implementation of mobile cognitive testing.

### Practitioners', Participants', and Caregivers' Technological Feedback

Results on feedback specific to the use of technology were mixed. There were some concerns regarding the older participants' ability to access telemedicine consultations or services. For example, Arighi et al. (7) identified that 34 participants were unable to respond to their scheduled televisit due to poor connection issues. Additionally, having a young caregiver did have a significant impact on televisit success (7). This is perhaps due to the fact that the caregiver is able to support the participant with any troubleshooting. However, most of the studies [i.e., (8, 10–12)] did praise the implementation of technologically mediated solutions. Iyer et al. (8) explored the opinions of older adults with varying levels of cognitive impairment (based on Functional Assessment Staging Tool [FAST] scores) through qualitative interviews and found that although clinicians found video technology services burdensome, patients and caregivers did not. In fact, patients reported feelings of connectedness and appreciated the discussions (8). Despite their initial disdain for arduous technological implementations, the service providers were appreciative of the ability to have many family members that were across geographically different areas united to discuss the patient's health (8).

Gately et al. (11) identified that caregivers valued video telemedicine services for their ability to greatly reduce travel needs (both issues with distance and travel but also facilitating dementia-related decreases in mobility and cognition), and increase the ability of family members with physical limitations or living far away to engage in medical visits. Zeghari et al. (10) contend that remote neuropsychological testing through their mobile unit and video chat system was a feasible endeavor. This was supported by scores on an accessibility scale in which participants considered the virtual call comparable to face-to-face meetings (10). Zamir et al. (12) revealed that telemedicine calls between care homes provided some residents with renewed energy and self-purpose.

### Barriers to Technological Adoption

Of the seven studies included in this review, four considered the potential barriers to technological adoption for older adults living with memory decline, Alzheimer's, and dementia (i.e., (7, 8, 11, 12). Gately et al. (11) noted several barriers, including the challenges of having discussions with older adults with cognitive issues over video (due to technical issues or natural decreases in focus/attention), potential difficulties for service providers in acquiring an accurate representation of the care recipient, etc. Arighi et al. (7) proposed that lack of access to a helpful caregiver may hinder the patient's ability to properly use the technology. Similarly, Iyer et al. (8) cite difficulties such as lack of technological literacy and devices with cameras. Zamir et al. (12) identified five themes (i.e., regaining sense of self and purpose, residents with dementia remember faces not technology, inter and intra connectedness, organizational issues creating barriers to long-term implementation, and situational loneliness to overcome) all of which may create long-term barriers to implementation.

## Discussion

This systematic review aimed to explore the use, feasibility, and acceptability of telemedicine applications for older adults with dementia during the COVID-19 pandemic. In this systematic review, we explored seven articles implementing various forms of telemedicine projects ranging from video and telephone calling [e.g., (3, 7, 10–12)], and modification of everyday technologies such as televisions [e.g., (9)]. The findings of this systematic review clarify noteworthy developments within telemedicine research in the wake of COVID-19 delivered to older adults with dementia [e.g., refinement of remote cognitive assessments through a mobile unit (10), or developing television-based treatments that are intuitively designed for older adults with dementia (9), etc.]. Two main themes were observed: *the barriers remaining to telemedicine implementation, in the wake of COVID-19* and *the benefits of telemedicine use during COVID-19*.

Notably, COVID-19 not only led to improvements in Internet-based services but was a strong catalyst that led to the dramatic, widespread adoption of telemedicine in healthcare systems worldwide, and somehow, this approach to care fit in the notoriously conservative healthcare industry, which is typically slow to adopt novel technologies (13–16). Indeed, the elderly have become one of the predominant demographics targeted for telemedicine projects as these devices have the possibility to connect, monitor, and assist seniors with healthcare professionals, emergency services, and family members across large distances without the need for in-person, face to face, interactions (17).

As the number of telemedicine projects continues to rise in response to the pandemic, it should be noted that the group for which the technology is perhaps most imperative (i.e., older adults with cognitive decline – most at-risk for COVID-19) may not be fully equipped to use it without the proper assistance (7, 11, 14). To begin with, the lack of knowledge and digital literacy are established causes of stress and disengagement with technology among older adults (18, 19). More so, older adults are typically less accustomed to technologies and may avoid them entirely (10). In this situation, a competent caregiver would play a crucial role (11). This distinction is further exemplified by Gately et al. (11), who affirm that without proper help and support, even individuals living with a mild form of dementia may have significant difficulties with using telemedicine services which would worsen as the disease progresses.

Arighi et al. (7) further noted the importance of caregiver assistance as a moderator for the success of their telemedicine intervention. Notably, it was found that when older patients received the support of younger caregivers (e.g., children or grandchildren), telemedicine consultations were significantly more successful (7). Zamir et al. (12) also posited that telemedicine approaches should be facilitated by younger care staff. The current systematic review revealed several barriers remaining within telemedicine practices applied to the geriatric population, specifically, individuals living with dementia, such as the inability to deal with technical issues, connectivity problems, as well as the loss of information due to being unable to properly examine the patient (7, 8, 11, 12).

Nevertheless, a consensus remains that telemedicine could positively impact patients and their access to healthcare [e.g., (8, 10–12)]. This suggests that despite the current issues that older adults with dementia may face, there remains an overall positive aspect to providing services via technology. Lai et al. (3) further propose the impact of their telemedicine intervention (via video conference) to help their older participants develop a stronger resilience to the effects of COVID-19 related isolation (as shown through improvements in the intervention groups QoL-AD scores).

Moreover, in all seven studies, the older adults showed a trend toward admiration for these technologies (3, 7–12). For example, chatting with physicians via video calls was welcomed [e.g., (3, 8–10)] and having access to telemedicine-based television services resulted in increased use of memory exercise game use (9). It may also indicate that older adults are willing to engage with new technologies if they recognize the benefits of using the device and have access to the technology. This is corroborated by Heinz et al.' (20) work, whereby it was proposed that seniors' motivation to engage with technology is higher when they are able to perceive the added benefits of technology use, such as increased autonomy and a better quality of life. Overall, these findings suggest that while there may be a concern regarding older adults' ability to use telemedical services, the benefits of digital interventions could outweigh this concern. This would be possible with proper design, support, oversight from caregivers and staff, in addition to providing a greater understanding of the usefulness of these tools to older adults.

Some limitations exist within the current set of studies. For instance, few quantitative mental health assessments were included. One such example is the study by Zamir et al. (12), in which they conclude that older adults' mental states were improved, as demonstrated through their qualitative analyses. Due to the limitations of qualitative research (e.g., lack of quantification of change), adopting the use of a mixed-method design in follow-up work can broaden the exploration and integration of these findings to provide a more complete interpretation of what the participants are experiencing (21, 22). Additionally, the majority of the telemedicine assessment or interventions that were identified were video conference-based. Finally, the current systematic review did not examine telemedicine practices occurring pre-pandemic. Future studies could perhaps examine the evolution and changes that telemedicine has undergone since the arrival of COVID-19 and examine individuals' experiences longitudinally.

## Conclusions

Based on the existing body of literature during COVID-19, telemedicine assessment and intervention approaches focusing on supporting older adults with dementia are presented as helpful tools. Despite the notable barriers that exist, such as those involving accessibility and digital literacy, it should be considered that telemedicine approaches and intervention remain a feasible alternative to connecting with individuals while reducing complications related to travel (e.g., difficulty moving, traffic, distance). Given the COVID-19 pandemic's international impact and the physical distancing and isolation measures it imposes, finding alternative ways to connect may continue to become more and more essential.

## Data Availability Statement

The original contributions presented in the study are included in the article/supplementary material, further inquiries can be directed to the corresponding author/s.

## Author Contributions

HS and SE conceived the presented idea and framework for the systematic review. SE conducted the search for articles in consultation with HS and made the table and completed the PRISMA diagram. SE and KC analyzed the articles in consultation with HS and drafted the manuscript. SE, KC, and HS were involved in the planning of the manuscript. KS, JG, CR, KB, KN, PL, KG, IV, and SR revised and provided feedback for the manuscript. All authors revised and approved the final manuscript.

## Conflict of Interest

SR receives a junior investigator salary award from the Fonds de Recherche Quebec–Santé and an investigator-initiated research grant from Satellite Healthcare (Dialysis Company) for an unrelated project. The remaining authors declare that the research was conducted in the absence of any commercial or financial relationships that could be construed as a potential conflict of interest.

## Publisher's Note

All claims expressed in this article are solely those of the authors and do not necessarily represent those of their affiliated organizations, or those of the publisher, the editors and the reviewers. Any product that may be evaluated in this article, or claim that may be made by its manufacturer, is not guaranteed or endorsed by the publisher.
